# Biological Activities and Phytochemicals of *Swietenia macrophylla* King

**DOI:** 10.3390/molecules180910465

**Published:** 2013-08-30

**Authors:** Soheil Zorofchian Moghadamtousi, Bey Hing Goh, Chim Kei Chan, Tara Shabab, Habsah Abdul Kadir

**Affiliations:** Biomolecular Research Group, Biochemistry Program, Institute of Biological Sciences, Faculty of Science, University of Malaya, Kuala Lumpur 50603, Malaysia; E-Mails: soheil.zorofchian@gmail.com (S.Z.M.); gbh-85@hotmail.com (B.H.G.); chimkei@gmail.com (C.K.C.); shbtara@gmail.com (T.S.)

**Keywords:** *Swietenia macrophylla*, mahogany, limonoids, biological activity, phytochemicals

## Abstract

*Swietenia macrophylla* King (Meliaceae) is an endangered and medicinally important plant indigenous to tropical and subtropical regions of the World. *S. macrophylla* has been widely used in folk medicine to treat various diseases. The review reveals that limonoids and its derivatives are the major constituents of *S. macrophylla*. There are several data in the literature indicating a great variety of pharmacological activities of *S. macrophylla*, which exhibits antimicrobial, anti-inflammatory, antioxidant effects, antimutagenic, anticancer, antitumor and antidiabetic activities. Various other activities like anti-nociceptive, hypolipidemic, antidiarrhoeal, anti-infective, antiviral, antimalarial, acaricidal, antifeedant and heavy metal phytoremediation activity have also been reported. In view of the immense medicinal importance of *S. macrophylla*, this review aimed at compiling all currently available information on its ethnomedicinal uses, phytochemistry and biological activities of *S. macrophylla*, showing its importance.

## 1. Introduction

Plants besides having the critical role of photosynthesis are also a source of numerous phytochemicals used through a variety of herbal remedies and foodstuffs with curative properties that help mankind sustain its health. Thereby, the development of pharmaceutical products necessitates a comprehensive investigation of medicinal plants to enhance our knowledge about their biological activities and the phytoconstituents responsible for them [1]. Additionally, the fact that only a limited number of medicinal plant species have received complete scientific inspection makes the need for comprehensive investigations in this area more evident [2]. Plants with a long history of use in ethno- medicine can be a rich source of substances for the treatment of various chronic or infectious diseases [3]. The big-leaved mahogany tree (*Swietenia macrophylla*), well known in the timber industry for its wood quality, is one such plant [4]. As *S. macrophylla* is important for the economy of many neotropical countries due to its high-quality timber [5,6], nuclear microsatellites and DNA-fingerprinting methods were established to verify the geographic origin of *S. macrophylla* in order to prevent illegal logging of this plant [7]. However, large areas of former *S. macrophylla* forests have been converted to other uses, and the remaining forests are few [8,9]. The depletion of *S. macrophylla* populations has led to concern for the future of the species and its commercial trade. In 2002, *S. macrophylla* was listed in Appendix II (species that may face extinction if trade is not controlled) of the Convention on International Trade in Endangered Species of Wild Fauna and Flora (CITES) [10]. Recent studies showed that *S. macrophylla*, besides its timber industry uses, also has noteworthy value and benefits in phytomedicine because of the variety of biological activities it presents. This review summarizes the botany, ethnomedicinal uses, phytochemistry, biological activities and mechanisms underlying the bioactivities of *S. macrophylla*.

## 2. Botany

### 2.1. Botanical Name

*Swietenia macrophylla* King.

### 2.2. Synonyms

*Swietenia belizensis* Lundell, *Swietenia candollei* Pittier, *Swietenia krukovii* Gleason, *Swietenia macrophylla* King var. *marabaensis* Ledouxet Lobato, *Swietenia tessmannii* Harms.

### 2.3. Common Names

Mahoni (all parts of Indonesia); baramahauni, bara-mahagoni, mahagni (Bangladesh); mahogany, big- or large-leaved mahogany, bastard mahogany, Brazilian mahogany tree, Colombian mahogany tree, Dominican mahogany, Honduras mahogany, Mexican mahogany tree, Peruvian mahogany tree, Spanish mahogany, West Indian mahogany (England); acajoudu Honduras, acajou du Venezuela, acajou étranger (France); Echtes mahagoni (Germany); mogano (Italy); cheria mahogany (Malaysia); mahok, mahonie (Netherland); mogno (Portugal); caoba, caoba de Honduras, caoba de Santo, caoba del Atlántico, caobahondureña, domingo, (Spain); mahokkani-baiyani, mahokkani-bailek (Thailand) [11,12].

### 2.4. Botanical Description and Distribution

The mahogany tree *Swietenia macrophylla*, also called “sky fruit” due to upward trend of its fruits towards the sky, is a beautiful, lofty, evergreen large tropical tree with a height of 30–40 m and girth of 3–4 m [13]. However, sometimes the height of this long-lived deciduous tree with an umbrella-shaped crown reaches a height of up to 50 m. *S. macrophylla* belongs to the Melicaceae family, with 50 genera and 1,400 species [14]. It grows natively throughout the tropical regions of the Americas, in particular Central and South American countries such as Mexico and Bolivia [15,16,17] and in west India, Malaysia, and southern China [18].

## 3. Ethnomedicinal Uses

*Swietenia macrophylla* has been used in Asia and many other countries to treat diverse ailments based on its antimicrobial, anti-inflammatory, antioxidant effects, antimutagenic, anticancer, antitumor and antidiabetic activities. Almost all parts of the plant are used in traditional medicine for the treatment of various human ailments. The fruit of *S. macrophylla* has been used commercially in health care products for the improvement of blood circulation and skin condition. The seed of *S. macrophylla* in particular has significant medicinal properties. In Malaysia, the seeds are used traditionally to treat hypertension, diabetes, and relieve pain [17]. The seeds of *S. macrophylla* have been reported to have anti-inflammatory, antimutagenicity and antitumor activity [19]. An Bolivian Amazonian ethnic group has used the seeds for leishmaniasis and as an abortion medicine [20]. In Indonesia, *S. macrophylla* seeds have been used as a folk medicine for treatment of diabetes, hypertension, and malaria [21]. The bark extract has been used as an astringent for wounds and used occasionally for tanning because of the rich red color if provides [22]. 

## 4. Phytochemistry

**Figure 1:**
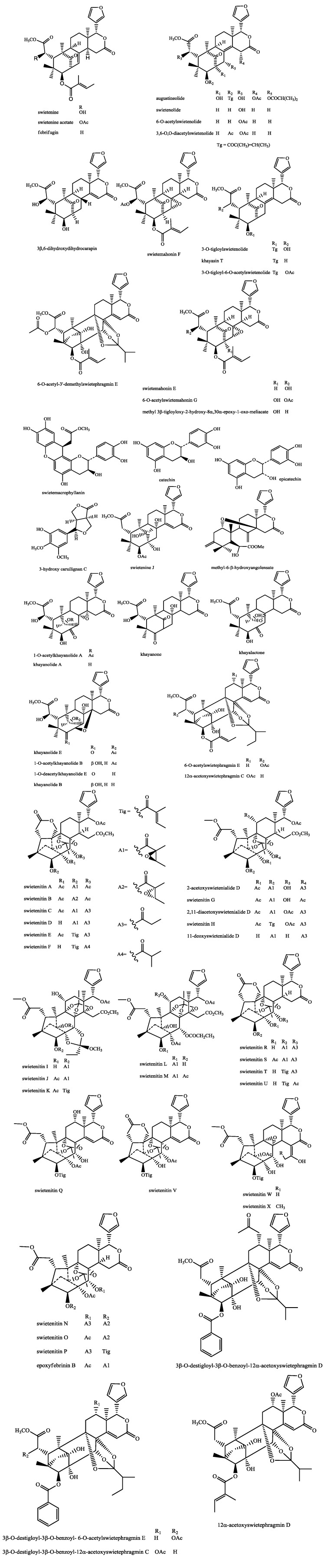
Chemical structures of various phytochemicals isolated from *S. macrophylla*.

Phytochemical investigations have shown that limonoids and their derivatives are the major constituents of *S. macrophylla* [23,24,25]. Limonoids are derived from tetracyclic triterpenes similar to euphol (H-20β) or tirucallol (H-20α) by a series of oxidative changes, interspersed with molecular rearrangements. Tetranortriterpenoids with a 4,4,8-trimethyl-17-furanyl steroidal skeleton is an alternative name for limonoids because in the process of oxidative changes, the side chain is eventually oxidised to a β-substituted furan ring by the loss of four carbon atoms [26]. During the last few years, there has been an increasing trend and awareness in *S. macrophylla* research. Quite a significant amount of research has already been carried out during the past few decades in exploring the chemistry of different parts of *S. macrophylla.* A wide array of isolated pure compounds with a plethora of pharmacological activities has been identified from different parts of *S. macrophylla*, as summarized in Table 1. The chemical structures of the major compounds isolated from this plant are presented in Figure 1.

**Table 1:** Compounds isolated from *Swietenia macrophylla*.

Plant part used	Compounds	Class	References
Seed	swietenine	Limonoid	[27]
	swietenolide	Limonoid	[23,28,29]
	8,30-epoxyswietenine acetate	Limonoid	
	swietenine acetate	Limonoid	
	swielenolidetiglate	Limonoid	
	swietenolidediacetate	Limonoid	[30,31]
	augustineolide	Limonoid	
	3β,6-dihydroxydihydrocarapin	Limonoid	[24]
	7-deacetoxy-7-oxogedunin	Limonoid	
	andirobin	Limonoid	
	proceranolide	Limonoid	[32]
	6-*O*-acetylswietenolide	Limonoid	[33,34]
	3,6-*O,O*-diacetylswietenolide	Limonoid	[35]
	khayasin T	Limonoid	
	swietemahonin E	Limonoid	[36]
	swietemahonin F	Limonoid	[21]
	swietemahonin G	Limonoid	
	2-hydroxyswietenine	Limonoid	
	6-deoxyswietenine (febrifugin)	Limonoid	[37]
	methyl 3β-tigloyloxy-2,6-dihydroxy-1-oxo-meliac-8(30)-enate	Limonoid	[25]
	methyl 3β-tigloyloxy-2-hydroxy-1-oxo-meliac-8(30)-enate	Limonoid	
	methyl 3β-tigloyloxy-2-hydroxy-8α,30α-epoxy-1-oxo-meliacate	Limonoid	
	methyl 3β-acetoxy-2,6-dihydroxy-8α,30α-epoxy-1-oxo-meliacate	Limonoid	
	methyl 3β-isobutyryloxy-2,6-dihydroxy-8α,30α-epoxy-1-oxo-meliacate	Limonoid	[38]
	6-*O*-acetyl-3′-demethylswietephragmin E	Limonoid	
	3-*O*-tigloylswietenolide	Limonoid	
	3-*O*-tigloyl-6-*O*-acetylswietenolide	Limonoid	[38,39]
	6-*O*-acetylswietemahonin G	Limonoid	[38,40]
	scopoletin	Coumarin	[38,41]
	stearic acid methyl ester	Fatty acid ester	
	β-sitostenone	Steroid	[38,42]
	3β-hydroxystigmast-5-en-7-one	Steroid	
	β-sitosterol	Steroid	[38,43]
	stigmasterol	Steroid	
Bark	swietemacrophyllanin	Polyphenol	[22]
	catechin	Polyphenol	[22,44]
	epicatechin	Polyphenol	[22,45,46]
Leaves	swietenolide monohydrate	Limonoid	[47]
	swietephragmin H	Limonoid	[48]
	swietephragmin I	Limonoid	
	swietephragmin J	Limonoid	
	swietemacrophine	Limonoid	
	*γ*-himachalene	Essential Oil	[49]
	germacrene D	Essential Oil	
	germacrene A	Essential Oil	
	cadina-1,4-diene	Essential Oil	
	hexadecanoic acid	Essential Oil	
	ethylhexadecanoate	Essential Oil	
	swietenine J	Limonoid	[50]
	methyl-6-β-hydroxyangolensate	Limonoid	[50,51]
	1-*O*-acetylkhayanolide A	Limonoid	[50,52]
	khayanolide E	Limonoid	[50,53]
	khayalactone	Limonoid	[50,54]
	khayanone	Limonoid	
	1-*O*-acetylkhayanolide B	Limonoid	[50,55]
	1-*O*-deacetylkhayanolide E	Limonoid	
	khayanolide A	Limonoid	
	khayanolide B	Limonoid	
	6-*O*-acetylswietephragmin E	Limonoid	[50,56]
	3*β*-*O*-destigloyl-3*β*-*O*-benzoyl- 6-*O*-acetylswietephragmin E	Limonoid	
	12α-acetoxyswietephragmin C	Limonoid	
	3*β*-*O*-destigloyl-3*β*-*O*-benzoyl-12α-acetoxyswietephragmin C	Limonoid	
	12α-acetoxyswietephragmin D	Limonoid	
	3*β*-*O*-destigloyl-3*β*-*O*-benzoyl-12α-acetoxyswietephragmin D	Limonoid	
Twig	swietenitin A	Limonoid	[57]
	swietenitin B	Limonoid	
	swietenitin C	Limonoid	
	swietenitin D	Limonoid	
	swietenitin E	Limonoid	
	swietenitin F	Limonoid	
	swietenitin G	Limonoid	
	swietenitin H	Limonoid	
	swietenitin I	Limonoid	
	swietenitin J	Limonoid	
	swietenitin K	Limonoid	
	swietenitin L	Limonoid	
	swietenitin M	Limonoid	
Twig	swietenitin N	Limonoid	[58]
	swietenitin O	Limonoid	
	swietenitin P	Limonoid	
	swietenitin Q	Limonoid	
	swietenitin R	Limonoid	
	swietenitin S	Limonoid	
	swietenitin T	Limonoid	
	swietenitin U	Limonoid	
	swietenitin V	Limonoid	
	swietenitin W	Limonoid	
	swietenitin X	Limonoid	
	2-acetoxyswietenialide D	Limonoid	
	2,11-diacetoxyswietenialide D	Limonoid	
	11-deoxyswietenialide D	Limonoid	
	epoxyfebrinin B	Limonoid	
Stem	3-hydroxycaruilignan C	Lignan	[59]

## 5. Biological Activities

### 5.1. Antiviral Activity

A bioactive compound, 3-hydroxycaruilignan C (3-HCL-C) was isolated from the most active ethyl acetate fraction of *S. macrophylla* stems and the chemical structure was identified using 1D and 2D nuclear magnetic resonance spectroscopy and confirmed using mass spectrometry. 3-HCL-C showed anti-HCV (Hepatitis C virus) activity at either the protein or RNA level at nontoxic concentrations, with an EC_50_ value of 10.5 ± 1.2 µM. The suppression of HCV RNA replication was increased by the combinations of 3-HCL-C, interferon-α (IFN-α), and HCV NS5B polymerase inhibitor (2′-C-methylcytidine; NM-107) or HCV NS3/4A protease inhibitor (telaprevir; VX-950). By inducing IFN-stimulated response element transcription and IFN-dependent antiviral gene expression, 3-HCL-C interfered with HCV replication and through this pathway it was demonstrated to be a potential antiviral agent [59].

### 5.2. Anti-Inflammatory Activity

Treatment of mice with *S. macrophylla* ethanolic seed extract (1,000 mg/kg body weight) reduced carrageenan-induced inflammation by 79%. The hexane and methanol fractions of *S. macrophylla* ethanol extract exhibited lower efficacies of 23% and 60% inflammatory inhibition, respectively [19]. In 2010 Chen and coworkers [38] isolated seventeen compounds by chromatographic purification of the ethyl acetate-soluble fraction of the *S. macrophylla* fruit methanolic extract on a silica gel column. 

An assay of the seventeen compounds on inhibition of superoxide anion generated by human neutrophils in response to fMet-Leu-Phe showed anti-inflammatory effects (IC_50_ ≤ 35.7 µM) for six limonoid compounds: 6-*O*-acetyl-3′-demethylswietephragmin E, 3,6-*O,O*-diacetylswietenolide, 3-*O*-tigloylswietenolide, 3-*O*-tigloyl-6-*O*-acetylswietenolide, swietemahonin E and 6-*O*-acetyl-swietemahonin G. The structure of the compounds was supported by ^1^H-^1^H COSY and NOESY experiments, and ^13^C-NMR assignments were confirmed by DEPT, HSQC, and HMBC techniques.

### 5.3. Anti-Infective Activity

Control of infections by *Pseudomonas aeruginosa* is critical and important due to the widespread antibiotic resistance reported for this pathogen [60]. The existence of anti-infective compounds in the seeds of *S. macrophylla* was proved by using a host-pathogen screening assay on *Caenorhabditis elegans* infected by *Pseudomonas aeruginosa* which is the common cause of nosocomial contamination in medical care facilities, leading to unwanted secondary infections in patients [61]. Although the ethyl acetate extract did not show any antibacterial activity *in vitro*, it enhanced the survival rate of the worms infected with *P. aeruginosa.* The extract boosted induction of gene pivotal for innate immunity of *C. elegans* such as defense gene *lys-7* which encodes for a lysozyme-like antimicrobial factor [62].

### 5.4. Anticancer and Antitumor Activity

The antitumor activity of the ethanol extract of *S. macrophylla* seeds and its hexane and methanol fractions were investigated using the Epstein-Barr virus early-antigen (EBV_EA) activation, with 12-0-tetradecanoylphorbol-13-acetate (TPA) as the tumor promoter. The results revealed considerable inhibitory activity on EBV_EA activation, indicating an antitumor-promoting effect [19]. The cytotoxic activity of the crude ethanol extract of the seeds of *S. macrophylla* and its fractions was assessed against selected human cancer cell lines, namely, HCT116, KB, Ca Ski and MCF-7 by using MTT assay. The *S*. m*acrophylla* ethyl acetate fraction (SMEAF) showed the most potent activity against HCT116 cell line (IC_50_ = 35.35 ± 0.50 μg/mL). The induction of apoptosis was confirmed both by DNA fragmentation using TUNEL assay and the externalization of phosphatidylserine using annexin V/PI staining. The cell cycle analysis revealed a prominent increase in sub-G1 population at concentrations of 0.05 mg/mL and above. Results also showed that SMEAF induced collapse of the mitochondrial membrane potential after 24 h and caused depletion in total intracellular glutathione [17].

### 5.5. Antimutagenic Activity

A micronucleus test was used for studying the antimutagenicity of the ethanol extract of *S. macrophylla* seeds. Treatment of mice with *S. macrophylla* ethanol seed extract (20 mg/kg body weight) reduced the number of micronucleated polychromatic erythrocytes induced by mitomycin C, a known mutagen, by roughly 50%. The result demonstrated a significant antimutagenicity of the *S. macrophylla* crude extract [19].

### 5.6. Antidiabetic Activity

Treatment of streptozotocin- and nicotinamide-induced type 2 diabetic rats with *S. macrophylla* seed methanol extract (300 mg/kg body weight) for 12 consecutive days reduced the fasting blood glucose level by 32.78% [63].The extract at the same dose also significantly reduced the elevated level of serum total cholesterol (18.56%) and triglyceride (10.41%), and increased the reduced liver glycogen level by 46.27% [63]. In oral glucose tolerance test (OGTT), the methanol extract of *S. macrophylla* seeds exhibited a 59.69% reduction in blood glucose level after 12 consecutive days of oral treatment with 300 mg/kg body weight demonstrating the potent antidiabetic activity of *S. macrophylla* [64]. 

Swietenine, a tetranortriterpenoid, was chromatographed from the ethanol soluble fraction of *S. macrophylla* seed extract by medium-pressure chromatography system employing gradient elution technique with hexane, chloroform (0–100%) and characterized by ^1^H- and ^13^C-NMR, MS and mp. This compound was found to possess considerable dose-dependent hypoglycemic effect in type 2 diabetic rats. Oral administration of swietenine (25 and 50 mg/kg body weight) significantly decreased fasting blood glucose level of type 2 diabetic rats in a dose-dependent manner. Treatment with swietenine significantly reduced the elevated cholesterol, triglyceride and liver glycogen level to the normal level in a dose-dependent manner when compared with non-diabetic control group [65]. Oral treatment with swietenine (25 and 50 mg/kg body weight) for 5 days reduced the fasting glucose level by 47.34 mg/dL and 55.85 mg/dL, respectively [65]. Oral administration of alcoholic seed extract of *S. macrophylla* and glibenclamide in streptozotocin-induced diabetic rats showed an improvement in body weight and decreased blood glucose level and the effect of *S. macrophylla* extract was more pronounced at 100 mg/kg body weight. Oral treatment with *S. macrophylla* extract and glibenclamide significantly increased the haemoglobin, glycosylated haemoglobin and serum insulin levels. The liver glycogen level in diabetic rats treated with *S. macrophylla* was elevated to the normal level obtained with glibenclamide [66]. Oral administration with the aqueous extract of *S. macrophylla* seeds (2 g/kg body weight) on streptozotocin-induced diabetic rats showed hypoglycaemic activity with a significant reduction of fasting plasma glucose by 98.66 ± 9.26 mg/dL [67]. 

### 5.7. Anti-Nociceptive Activity

Oral treatment of mice with ethanol and aqueous extracts of *S. macrophylla* fruits (200 mg/kg body weight) showed a reduction in the number of writhing, tail immersion and hot plate response exhibiting a powerful anti-nociceptive activity. Tail-flick and hot-plate responses at the dose of 200 mg/kg body weight also corroborated the considerable analgesic effect of the ethanol extract of *S. macrophylla*. The analgesic activity produced through a mechanism partially connected to either lipooxygenase and/or cyclooxygenase via the arachidonic acid cascade and/or opioid receptors producing analgesia in thermal and chemical pain models. The total results confirmed anti-nociceptive activity of *S. macrophylla* and substantiated its traditional consumption as pain killer [68].

### 5.8. Hypolipidemic Activity

Oral treatment with swietenine (25 and 50 mg/kg body weight) in neonatal-streptozotocin induced type 2 diabetic rats significantly reduced the elevated cholesterol and triglyceride level. After 5 days of treatment with swietenine (25 mg/kg body weight) the level of cholesterol and triglyceride was reduced by 17.25 mg/dL and 29.12 mg/dL, respectively. For 50 mg/kg of swietenine, this reduction increased to 24.35 mg/dL and 31.58 mg/dL, respectively [65]. The treatment with the methanol extract of *S. macrophylla* (300 mg/kg body weight) in streptozotocin- and nicotinamide-induced type 2 diabetic rats for 12 consecutive days caused reduction in the elevated level of serum total cholesterol (18.56%) and triglyceride (10.41%), respectively [63]. Under the same experimental condition the extract showed 45.41% and 37.78% reduction in cholesterol and triglyceride levels in streptozotocin-induced diabetic rats, respectively [64]. 

### 5.9. Antioxidant Activity

Three compounds, namely, catechin, epicatechin, and swietemacrophyllanin were isolated from the fractionation of the acetone extract of *S. macrophylla* bark with *n*-hexane, diethyl ether and ethyl acetate, followed by subsequent chromatographic separation of the fractions*.* The structure of the compounds was elucidated by spectroscopic data and by comparison of the NMR data with those of catiguanins A and B, phenylpropanoid-substituted epicatechins [22]. These compounds from the polyphenols or flavan-3-ols class exhibited antioxidant activity using the DPPH [1,1-diphenyl-2-picrylhydrazyl] free radical scavenging assay. The study showed that swietemacrophyllanin had the strongest antioxidant activity, with an IC_50_ value of 56 µg/mL compared with Trolox used as a reference. The ethanol extract of *S. macrophylla* seeds also showed antioxidant activity in the streptozotocin-induced diabetic rats. Antioxidants such as vitamins C and E levels in the plasma, and reduced glutathione level in the plasma, kidney, and liver increased in rats treated with the extract [69].

### 5.10. Antimicrobial Activity

The antibacterial and antifungal efficacy of the methanol and aqueous extracts of *S. macrophylla* seeds was evaluated on selected pathogenic bacterial and fungal strains by disc diffusion and micro dilution assay methods. The methanol and aqueous extract of *S. macrophylla* exhibited activity against *Pseudomonas aeruginosa* MTCC 424, *Klebsiella pneumonia* MTCC 109, *Bacillus cereus* MTCC 430, *Staphylococcus aureus* MTCC 96, and *Escherichia coli* MTCC 443 [70]. The extracts showed antifungal activity against *Candida albicans* MTCC 183, *Aspergillus niger* MTCC 16404, *Cryptococcus albidus* MTCC 2661, and *Aspergillus flavus* MTCC 1973. *E. coli* MTCC 443 and *C. albidus* MTCC 2661 were found to be the most susceptible bacteria and fungi, respectively. The methanol extract demonstrated greater efficacy than aqueous extract of *S. macrophylla*. The results showed that the seeds of *S. macrophylla* possessed marked antibacterial and antifungal activity [70].

### 5.11. Antidiarrhoeal Activity

The petroleum ether extract of *S. macrophylla* seeds was investigated for antidiarrhoeal effect in Wister albino rats and the results showed a significant antidiarrhoeal activity proved by the reduction in the rate of defecation and consistency of faeces in castor oil induced diarrhoea rats at various extract doses of 25, 50 and 100 mg/kg body weight. A reduction of 4.45% to 34.60% in intestinal transit and significant inhibition of castor oil induced enterpooling exhibited profound anti-diarrhoea activity of the extract. The *in vivo* study revealed that seeds of *S. macrophylla* may be a source of antidiarrhoeal drug in the future [71]. 

### 5.12. Antimalarial Activity

A decoction of *S. macrophylla* seeds was reported as an antimalarial remedy [21]. The antimalarial effect of the *S. macrophylla* seed methanol extract was investigated against *Plasmodium falciparum* [72]. The bark extract of *S. macrophylla* showed strong antimalarial activity (78% inhibition at 100 µg/mL) *in vitro* on chloroquine resistant *P. falciparum* strain (Indo). The *in vivo* study of the bark extract at 250 mg/kg body weight exhibited 73% inhibition on the rodent malaria *P. vinckeipetteri* 279BY [73]. The aqueous extract of *S. macrophylla* seeds showed antibabesial activity besides the antimalarial activity against *P. falciparum* [74].

### 5.13. Antifeedant Activity

In an antifeedant study, four out of fifteen limonoids isolated from the acetone extract of *S. macrophylla* fruits washed with petroleum ether (60–80 °C) and triturated with ethyl acetate for silica gel column chromatography using CHCl_3_ with increasing amount of ethyl acetate, showed good antifeedant activity against *Spodoptera frugiperda*. Swietenolide, 6-*O*-acetylswietenolide, 3,6-*O*,*O*-diacetylswietenolide, and swietemahonin F were subjected to a bioassay on the final instar larvae of *S. frugiperda* at the concentration of 1,000 ppm. The results revealed that swietenolide exerted the greatest antifeedant activity (antifeedant index of 94.1 ± 2.90). The other compounds; 6-*O*-acetylswietenolide, 3,6-*O*,*O*-diacetylswietenolide, and swietemahonin F, exhibited antifeedant indices of 72.2 ± 19.60, 72.0 ± 9.38, and 70.2 ± 8.90, respectively. The compounds were identified by detailed analysis of their respective high resolution ^1^H- and ^13^C-NMR spectra including COSY, HETCOR and HMBC correlations [32].

### 5.14. Acaricidal Activity

Varroatosis is a disease caused by *Varroa destructor* mites (Acari: Varroidae) which has become a serious pest problem for honeybees, *Apis mellifera* and *Apis cerana* (Hymenoptera: Apidae) worldwide. The mites cause damage to immature and adult bees by feeding on hemolymph, thus, greatly weakening or killing the bees [75]. The ethanol extract of the stem bark and leaves of *S. macrophylla* showed acaricidal activity in the honeybee colonies infested by *V. destructor* mites. The rate of infestation after 12 days of treatment with 500 ppm of *S. macrophylla* bark and leaf extract decreased to 1.08% and 2.41%, respectively. These extracts, which proved to be safe to the environment and harmless to the bees, are promising as safe natural products for the control of *Varroa* mites [76]. 

### 5.15. Heavy Metal Phytoremediation Activity

In the study on the phytoremediation potential of *S. macrophylla*, hydroponic experiments were performed with cadmium concentration gradients at concentrations of 0, 7.5, 15, and 30 mg/L to identify cadmium accumulation and tolerance of *S. macrophylla* seedlings as well as their potential for phytoextraction. The results indicated that cadmium inhibited *S. macrophylla* seedling growth at the highest exposure concentration of 30 mg/L and demonstrated great potential for phytoextraction at concentrations of 7.5 and 15 mg/L. *S. macrophylla* seedlings accumulated up to 154 mg/L cadmium in twigs at the concentration of 15 mg/L. These results suggest that *S. macrophylla* can be used as a potential candidate for remediating cadmium contaminated sites in tropical regions, because of cadmium uptake capacity and high biomass production of mahogany shoots [77,78].

## 6. Conclusions

Besides being a good source for timber products and reforestation programmes in the tropics, *S. macrophylla* exhibits a wide spectrum of significant biological activities. A review of the phytochemistry of *S. macrophylla* has revealed a large number of limonoids, the principle bioactive compounds, which have not been investigated for their bioactivities. In view of the medicinal importance of *S. macrophylla* greater attention is warranted towards the discovery of their potential pharmaceutical uses.
